# Interim Results of a Multicenter Trial with the New Electronic Subretinal Implant Alpha AMS in 15 Patients Blind from Inherited Retinal Degenerations

**DOI:** 10.3389/fnins.2017.00445

**Published:** 2017-08-23

**Authors:** Katarina Stingl, Ruth Schippert, Karl U. Bartz-Schmidt, Dorothea Besch, Charles L. Cottriall, Thomas L. Edwards, Florian Gekeler, Udo Greppmaier, Katja Kiel, Assen Koitschev, Laura Kühlewein, Robert E. MacLaren, James D. Ramsden, Johann Roider, Albrecht Rothermel, Helmut Sachs, Greta S. Schröder, Jan Tode, Nicole Troelenberg, Eberhart Zrenner

**Affiliations:** ^1^Centre for Ophthalmology, University of Tuebingen Tuebingen, Germany; ^2^Retina Implant AG Reutlingen, Germany; ^3^Nuffield Laboratory of Ophthalmology, Oxford Eye Hospital, Oxford University Hospitals NHS Foundation Trust, University of Oxford Oxford, United Kingdom; ^4^Department of Ophthalmology, Katharinenhospital, Klinikum Stuttgart Stuttgart, Germany; ^5^Städtisches Klinikum Dresden Friedrichstadt, University Teaching Hospital Dresden, Germany; ^6^Division Pediatric Otorhinolaryngology and Otology - Olgahospital, Department of Otorhinolaryngology, Klinikum Stuttgart Stuttgart, Germany; ^7^Department of Ophthalmology, University of Kiel Kiel, Germany; ^8^Institute for Microelectronics, University of Ulm Ulm, Germany; ^9^Werner Reichardt Centre for Integrative Neuroscience, University of Tuebingen Tuebingen, Germany

**Keywords:** subretinal implant, RETINA IMPLANT Alpha AMS, neuroprosthetics, retinitis pigmentosa, artificial vision, hereditary retinal disease, photoreceptor degeneration

## Abstract

**Purpose:** We assessed the safety and efficacy of a technically advanced subretinal electronic implant, RETINA IMPLANT Alpha AMS, in end stage retinal degeneration in an interim analysis of two ongoing prospective clinical trials. The purpose of this article is to describe the interim functional results (efficacy).

**Methods:** The subretinal visual prosthesis RETINA IMPLANT Alpha AMS (Retina Implant AG, Reutlingen, Germany) was implanted in 15 blind patients with hereditary retinal degenerations at four study sites with a follow-up period of 12 months (www.clinicaltrials.gov NCT01024803 and NCT02720640). Functional outcome measures included (1) screen-based standardized 2- or 4-alternative forced-choice (AFC) tests of light perception, light localization, grating detection (basic grating acuity (BaGA) test), and Landolt C-rings; (2) gray level discrimination; (3) performance during activities of daily living (ADL-table tasks).

**Results:** Implant-mediated light perception was observed in 13/15 patients. During the observation period implant mediated localization of visual targets was possible in 13/15 patients. Correct grating detection was achieved for spatial frequencies of 0.1 cpd (cycles per degree) in 4/15; 0.33 cpd in 3/15; 0.66 cpd in 2/15; 1.0 cpd in 2/15 and 3.3 cpd in 1/15 patients. In two patients visual acuity (VA) assessed with Landolt C- rings was 20/546 and 20/1111. Of 6 possible gray levels on average 4.6 ± 0.8 (mean ± *SD, n* = 10) were discerned. Improvements (power ON vs. OFF) of ADL table tasks were measured in 13/15 patients. Overall, results were stable during the observation period. Serious adverse events (SAEs) were reported in 4 patients: 2 movements of the implant, readjusted in a second surgery; 4 conjunctival erosion/dehiscence, successfully treated; 1 pain event around the coil, successfully treated; 1 partial reduction of silicone oil tamponade leading to distorted vision (silicon oil successfully refilled). The majority of adverse events (AEs) were transient and mostly of mild to moderate intensity.

**Conclusions:** Psychophysical and subjective data show that RETINA IMPLANT Alpha AMS is reliable, well tolerated and can restore limited visual functions in blind patients with degenerations of the outer retina. Compared with the previous implant Alpha IMS, longevity of the new implant Alpha AMS has been considerably improved. Alpha AMS has meanwhile been certified as a commercially available medical device, reimbursed in Germany by the public health system.

## Introduction

Inherited retina degenerations (IRD) are a broad group of genetic retinal disorders of varying severity with retinitis pigmentosa being the most common form. Its estimated prevalence is between 1 in 3,000–7,000 (Ferrari et al., 2011). Most of these retinal degenerations lead to a progressive visual loss and eventually to blindness caused by photoreceptor degeneration and atrophy. As these diseases are genetically heterogeneous, a causative cure is not yet available. However, several approaches tested in clinical trials in the last 10 years are close to, or have reached, the state of approval in the USA or Europe. Gene replacement therapy has shown positive effects for several genes (Bainbridge et al., 2008; MacLaren et al., 2014; Bennett et al., 2016), electrostimulation might be an option to delay progress for some retinitis pigmentosa patients (Schatz et al., 2011, 2017) and retinal implants have shown to restore measurable vision in some blind patients sufficient for object localization and rough details detection (Zrenner et al., 2011; Humayun et al., 2012; Ayton et al., 2014; Stingl et al., 2015). Moreover, further alternative approaches are being tested clinically and pre-clinically worldwide such as pharmacotherapy, stem cell research or optogenetics (Busskamp et al., 2012; Singh et al., 2013; Scholl et al., 2015).

Today three different types of retinal implants are commercially available in the USA and/or Europe: the epiretinal prosthesis devices Argus® II (Second Sight, Sylmar, California) and IRIS® II (Pixium Vision, Paris, France) and the subretinal device RETINA IMPLANT Alpha AMS (Retina Implant AG, Reutlingen, Germany). It should be noted that the epiretinal prosthesis devices are transmitting visual information via electrical stimulation of the ganglion cells, whereas the subretinal approach aims to replace the degenerated photoreceptors and is stimulating the bipolar cells of the remaining inner retina. Thus, patients blind because of damage of the inner retina (e.g., glaucoma) or other diseases which cause more than degeneration of photoreceptors cannot benefit from the RETINA IMPLANT Alpha AMS. Efficacy results from 29 blind patients wearing the RETINA IMPLANT Alpha IMS device—a precursor of Alpha AMS—have been published recently (Stingl et al., 2015). The results showed that the earlier version of the implant, the RETINA IMPLANT Alpha IMS is able to restore low but useful vision in patients blind from hereditary degenerations of the photoreceptors with visual acuities up to 20/546, as measured by standardized Landolt C-rings, and 45% of patients could recognize object shapes or rough details in everyday life. Despite promising functional results, the durability and life-time of the device was not optimal, mainly due to technical failures which occurred in some implants within the 12 month clinical trial observation period. Some factors leading to these technical problems have already been solved for RETINA IMPLANT Alpha IMS by adapting surgical techniques to minimize the mechanical stress onto the cable and thus preventing breaks of the intraorbital cable (Kernstock et al., 2011). However, only further technical development of the device, resulting in the new version RETINA IMPLANT Alpha AMS, could increase the life-time of the device significantly, due to improved materials, and design (Daschner et al., 2017).

Here we present the functional results of 15 blind patients due to a hereditary retinal disease who received the RETINA IMPLANT Alpha AMS in one eye, at four study sites during two related clinical trials.

## Materials and methods

### Patient recruitment

In three study sites in Germany (Tuebingen, Kiel, Dresden, www.clinicaltrials.gov NCT01024803) and one site in Great Britain (Oxford, www.clinicaltrials.gov NCT02720640) patients with end-stage of a hereditary retinal degeneration were recruited for the participation in the clinical trial. Patients with rod-cone and cone-rod degenerations, as well as choroideremia, who were not able to use their remaining vision for localization of objects, self-sustained navigation and orientation (impaired light localization or worse) were eligible for participation.

Further inclusion criteria were: age between 18 and 78 years, pseudophakia in the study eye, an adequate retinal perfusion of the macular region (target implantation area) and ability to read normal print in earlier life, optically corrected without magnifying glass.

Ophthalmologic exclusion criteria were a period of appropriate visual functions <12 years, Optical Coherence Tomography (OCT) findings of retinal edema and/or scar tissue within target region for implant as well as absent layering of the inner retina, heavy clumped pigmentation at posterior pole or any other ophthalmologic disease with relevant effect upon visual function (e.g., glaucoma, optic neuropathies, trauma, diabetic retinopathy, retinal detachment, amblyopia). Additionally, systemic diseases that might imply considerable risks with regard to the surgical interventions and anesthesia, neurological and/or psychiatric diseases, hyperthyroidism or hypersensitivity to iodine and pregnancy/nursing, and participation in another interventional clinical trial within the past 30 days were exclusion criteria.

### RETINA IMPLANT alpha AMS

**Figure 1:**
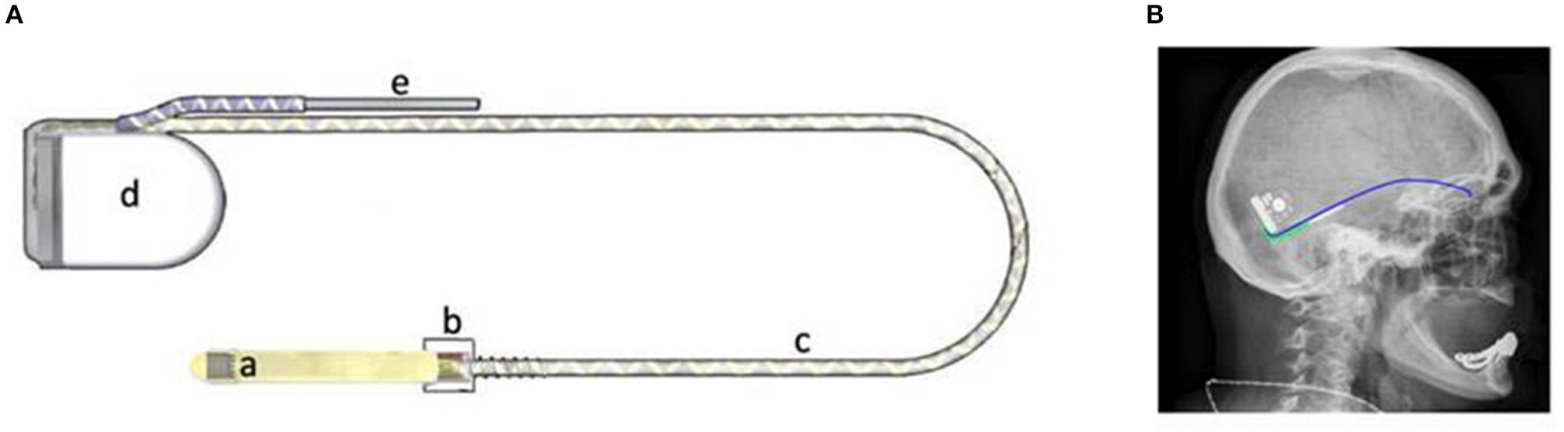
**(A)** Subretinal Implant RETINA IMPLANT Alpha AMS with (a): Polyimide foil and attached CMOS chip; (b): Sclera patch on ceramic chip; (c): Silicone cable; (d): Subdermal ceramic housing; (e): Return electrode. **(B)** View of silicone-power-cable routed on top of skull bone (below periost) and return electrode at temple area.

The subretinal implant RETINA IMPLANT Alpha AMS (Figure 1, internal parts) consists of a metal–oxide–semiconductor (CMOS) chip (Rothermel et al., 2009) attached to a distal polyimide (PI) foil, which are the only sub-retinal parts of the implant within the eye (Figure 1Aa). The 4.0 mm × 3.2 mm × 70 μm sized chip encompasses 1,600 pixel cells. Each pixel cell has a dimension of 70 × 70 μm and includes a photodiode, an amplifier and a stimulation electrode. The chip is subretinally implanted between the retina and the pigment epithelium and each pixel cell on the chip surface stimulates the adjacent bipolar cells according to the local light intensity measured by the photodiode. A detailed description of the conversion of light to electrical energy has been published for the Alpha IMS, but the principle is essentially the same for the Alpha AMS (Stingl et al., 2016). RETINA IMPLANT Alpha AMS stimulates the remaining retinal cells with biphasic current and voltage controlled pulses (cathodic phase first) and both the pulse duration and the frequency can be adjusted to each patient's individual needs in the range of 0.1–2.0 ms for each phase and 0.5–500 Hz, respectively. The required energy to power the 1,600 individual amplifiers in the pixel cells reaches the chip via a polyimide foil in the eye and a silicone power cable (Figures 1AC), which runs under the temporal muscle (Figure 1B). The silicone power cable is connected to a ceramic housing behind the ear (Figure 1Ad). This ceramic housing contains a magnet, a coil for inductive coupling, and electric circuits to generate the desired currents. The patient carries an external power supply, which also allows adjustment of the chip sensitivity and brightness to the local light conditions. The power supply's external coil is on a coaxial cable and attaches transdermally and magnetically to the implanted ceramic housing behind the ear.

The RETINA IMPLANT Alpha AMS is an advanced version of the previous Alpha IMS device; the main changes are summarized in Table 1.

**Table 1:** This table summarizes some of the changes made in device design of the RETINA IMPLANT Alpha IMS, leading to the new version RETINA IMPLANT Alpha AMS with similar efficacy outcome but improved life-time.

	**RETINA IMPLANT Alpha IMS**	**RETINA IMPLANT Alpha AMS**	**Reason for change**
Chip supplier	Institut für Mikroelektronik Stuttgart (IMS CHIPS)	Ams AG, Austria	Industrial chip manufacturing
Stimulation pulse/Type	Monophasic anodic/Voltage pulse (0.0–2.0 V)	Biphasic cathodic first/Current and Voltage controlled pulse (± 1.2 V)	Optimized charge transfer
Electrode material	Iridium, anodic oxidation	Sputtered Iridium Oxide	Increase of charge injection capability
Electrode shape/Dimension	Square, 50 × 50 μm	Round, diameter 30 μm	Increase of long-term stability
Electrode number	1,500	1,600	Full 40 × 40 pixel array
Chip size	3.2 × 3.1 mm	3.2 × 4.0 mm	Additional area for electronic circuits and bond contacts
Foil substrate		Floating traces between conductive traces	Increase of long-term stability
Silicone cable		Shortened, closely coiled cable part was increased	Increase of long-term stability

### Inclusion/exclusion criteria and surgery

Inclusion and exclusion criteria were essentially analogous in both studies (see Section Patient Recruitment).

Details of the surgical implantation procedure have been described previously (Besch et al., 2008; Sachs et al., 2010; MacLaren, 2017).

### Study procedures

Treatment duration was not specified in either study, as the implant is intended to remain in the eye as long as it provides benefit to the patient. The follow up duration was 12 months post-implantation surgery for both studies. The device was activated for the first time ~1 month after surgery. Subsequent follow-up visits were done 2, 3, 6, 9, and 12 months after surgery. As both studies are not completed at the time of submission of this manuscript, some patients have not yet completed the study (as indicated in **Figure 7**).

The primary efficacy endpoint of both trials was significant improvement in activities of daily living (ADL) with implant-ON vs. OFF, as assessed via ADL tasks and recognition tasks. Secondary efficacy endpoints in both studies were significant improvement of visual acuity/light perception and/or object recognition. All tests have been described previously in detail (Zrenner et al., 2011; Stingl et al., 2013, 2015); a short description is given below.

Additional endpoints of the studies covered patient safety and included assessment of damage of structures and function that had been functional before surgery, damage to health and/or wellbeing of patients, stability of implant function and stability of body structure and function related to implant system. A detailed report regarding the safety outcome of the studies will be published after completion of the studies.

#### Activities of daily living tasks

ADL tasks include tasks performed on a black table using white objects. Three different table tasks were performed:

#### Activities of daily living tasks—shapes

The first task used four geometrical shapes of about 5° visual angle each (e.g., triangle, square, circle, rectangle). The patient was asked how many objects could be seen, where they were and what they were. Correct responses were documented as scores (from 0 to 4 for each of the three questions).

#### Activities of daily living tasks—table

The second task used four dining objects (e.g., cup, cutlery) which were placed around a large white plate serving as reference for the patient. The patient was asked how many objects could be seen, where they were and what they were. Correct responses were documented as scores (from 0 to 4 for each of the three questions).

#### Activities of daily living tasks—eye-hand coordination

To test the eye-hand coordination, a large (~20° visual field) white ring or square frame was placed on the table and the patient was given either a chess piece or a small white egg cup. The patient was asked to localize the ring/square visually and place the chess piece/egg cup inside the frame. The test was counted as passed if the patient managed to place the object inside the frame without it standing on the frame itself.

#### Recognition of grayscales

For assessment of grayscales, the screen (as used in BaLM test, see below) was divided in half with one side always showing a gray area with 50% of the screen's brightness. The other side showed a gray value of 0% (white), 25, 37.5, 50, 62.5, 75, or 100% (black) with minimal and maximal brightness of typically 8 and 200 cd/m^2^, respectively. Each of the combinations was tested three times in a random order and the patient had to determine whether there was a difference in brightness and if so, which side was brighter. For the subsequent analysis, the 50/50% (identical gray levels) was not included. For the other six combinations a grayscale was counted as correctly discerned if 2 out of 3 repetitions were correctly distinguished.

#### Basic light and motion test

Light threshold perception, light source localization, and motion detection of dot patterns were tested on a screen, viewed at 60 cm, as either 2- or 4-alternative forced-choice (AFC) tests of 8 trials each (Basic Light and Motion—BaLM test; Bach et al., 2010). The participants responded via a keyboard or verbally. A test was considered as “passed”, if 75% (2AFC) or 62.5% (4AFC) of the answers were correct. It should be noted that the original test was developed with 24 trials, resulting in a probability of reaching or exceeding the “passed” criterion by chance of 1.1 and 0.011%, respectively. As 24 trials proved to be too tiring for many patients in previous studies the number of trials was reduced to eight, which resulted in a probability for a false positive of 14.5% (2AFC) and 2.7% (4AFC) in an individual test run, based on achieving 6 ormore out of 8 (2AFC) or 5 ormore out of 8 (4AFC) correct answers.

#### Basic grating acuity and visual acuity

Detection of gratings of different spatial frequencies were measured via the BaGA test (Wilke et al., 2007) and visual acuity (VA) using standardized Landolt C-rings in contrast reversal (white ring on black background), tested on a screen and viewed at 60 cm as either 2- or 4-alternatives forced-choice tests of 8 or 12 trials per resolution level. The participant was asked to tell the orientation of the grating and the direction of the C-ring gap, respectively. The participants responded via a keyboard or verbally.

#### Statistics

Missing data was not included in any analyses and extrapolation or last observation carried forward (LOCF) was not implied. Statistical analyses were performed with the software JMP, version 13.0.0 (SAS Institute Inc.). All tests performed were non-parametric, given the small sample size and thus the inadequacy to test for normal distribution. For assessments which were graded (ADL tasks shapes and table set-up) the Wilcoxon test with paired data was used, analyzing the difference between scores with implant ON vs. implant OFF. Patient data where only one condition was present (e.g., either ON or OFF) was omitted from the analysis. For analyses of assessments which were graded as “passed” or “failed,” the Fisher's exact test was used. An adjustment for multiple testing was not done for any of the analyses performed.

## Results

In total, 21 patients were screened for RETINA IMPLANT Alpha AMS. The reasons for exclusion after screening were as follows: pre-existing glaucoma, suspected non-functionality of the inner retina, remaining visual function allowing shape detection, macular edema, and heavy clumped pigmentation at posterior eye pole. One patient withdrew consent prior to the implantation.

Ten participants (6 female, 4 male) from three sites in Germany (Tuebingen, Kiel, Dresden, www.clinicaltrials.gov NCT01024803) and five participants (4 females, 1 male) from Great Britain (Oxford, www.clinicaltrials.gov NCT02720640) with a total mean age (± *SD*) of 55.2 ± 10.2 years between 34 and 70 years received the RETINA IMPLANT Alpha AMS in one eye. Fourteen of the participants had retinitis pigmentosa and one had cone-rod dystrophy. Visual function prior to implantation was light perception without projection (fourteen participants) or no light perception (one participant). None of the participants had any other eye diseases that might have affected the visual pathway.

No obvious difference in implant-mediated visual perception was seen in the one patient with the cone-rod degeneration as compared to the other patients.

Implant-mediated visual perception was observed in 13/15 patients. In one patient (RIAG-DD-02) the implant was probably damaged slightly during implantation and did not function correctly. In another patient (OX-RI-04) a combination of damage to the connecting foil and incorrect implantation procedure resulted in a non-functional chip. The chip was subsequently replaced successfully in an exchange procedure, although this was an “off-study” procedure as the patient was no longer part of the formal clinical trial. The results of the remaining 13 patients for each of the particular tests are given in the following paragraphs. A summary of the best results achieved with the implant switched ON for each individual patient is shown in Table 2. Please note that patient RIAG-KI-04 performed well in the ADL tasks at month 3, but as no light perception or light localization test was done at this visit, a “(−)” is noted in Table 2 for both tests.

**Table 2:** This table summarizes the best results achieved for each patient with a functional implant with the implant switched ON.

**Patient-ID**	**Light**	**Location**	**Motion**	**Eye-hand coordination**	**Basic grating acuity (cpd)**	**Landolt C**	**Grayscales (X/6 levels)**
RIAG-TU-16	+	+	−	+	0.66	−	4/6
RIAG-TU-18	+	+	−	+	0.33	−	4/6
RIAG-TU-20	+	+	−	−	0.33	−	–
RIAG-TU-21	+	+	+	+	1	20/546	5/6
RIAG-TU-23	+	+	−	+	0.66	20/1111	3/6
RIAG-TU-24	+	+	+	+	0.1	−	5/6
RIAG-KI-03	+	+	−	+	0.1	−	–
RIAG-KI-04	(−)	(−)	−	+	-	−	–
RIAG-DD-04	+	+	−	+	0.1	−	5/6
OX-RI-01	+	+	−	+	3.3	−	5/6
OX-RI-02	+	+	−	+	1	−	6/6
OX-RI-03	+	+	−	+	0.1	−	4/6
OX-RI-05	+	+	−	+	0.33	−	5/6

*For all tests “+” indicates a successful passing of the test and “−” indicates that the patient either failed to perform the task or that this test has not been done because one or more of the previous tests considered easier were failed as well. “(−)” is indicated for patient RIAG-KI-04 for both the light perception and light localization test as the patient passed the ADL table tasks and it can thus be assumed that these visual functions were present, but they were not tested. Basic grating acuity (grating orientation detection) results are shown in cycles per degree (cpd), the visual acuity as tested by Landolt C rings in the Snellen fraction. For gray levels the number of correctly distinguished shades (X/6 levels, a gray level was deemed correctly distinguished if the patient discerned 2 out of 3 repetitions correctly) is shown*.

### Activities of daily living tasks

**Figure 2:**
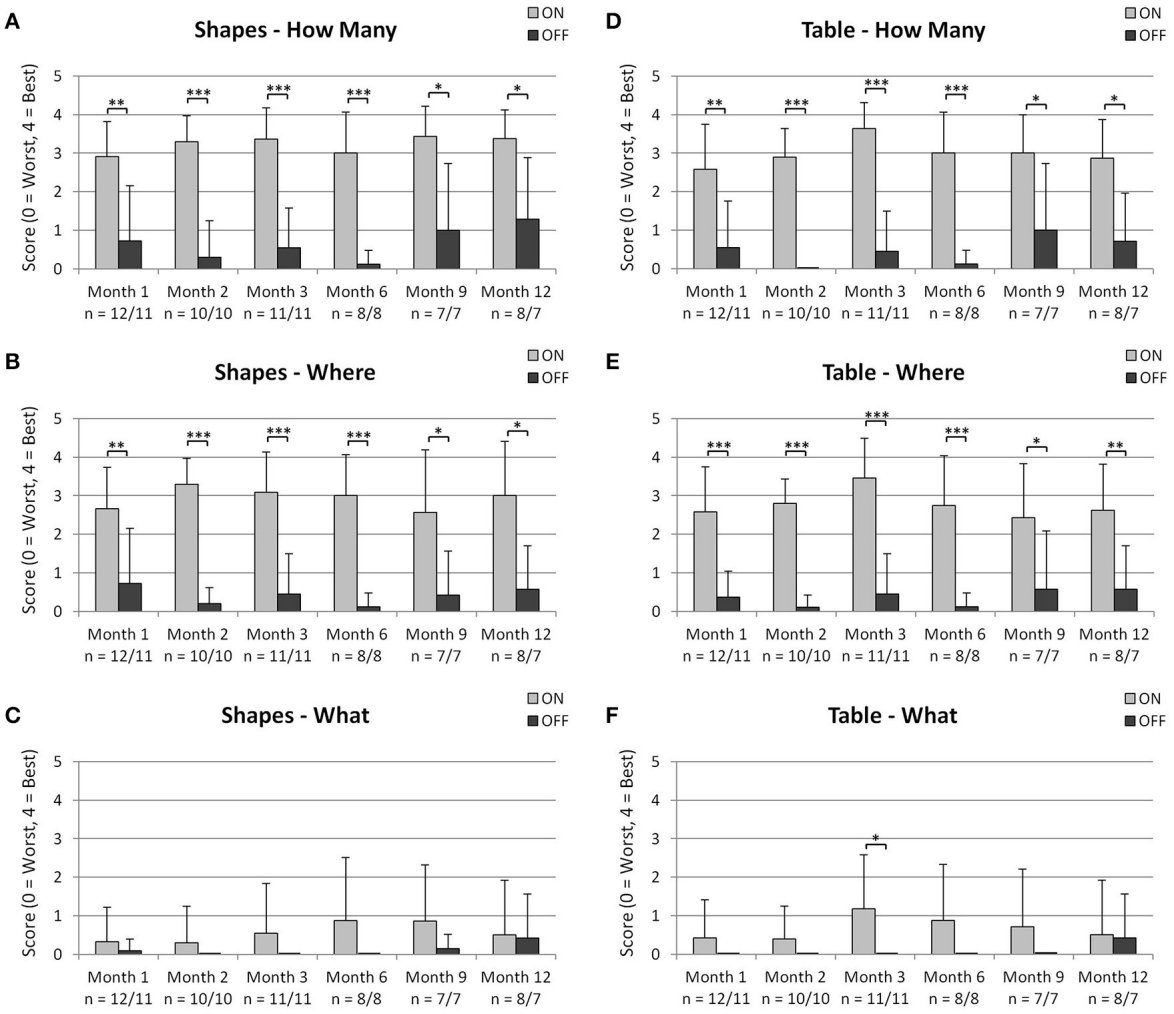
Summary (mean + *SD*) of scores for the ADL tasks shapes **(A–C)** and table set-up **(D–F)** for all time points tested (month 1, 2, 3, 6, 9, 12) for the conditions implant ON vs. implant OFF. **(A,D)** show the scores for detection of the item on the table, **(B,E)** show the scores for the localization of the item and **(C,F)** show the scores for recognition of the item. The number of participants with available data is indicated below the x-axis for both implant ON/implant OFF. Significant differences as analyzed via the Wilcoxon test are shown in the figures with ^*^p < 0.05, ^**^p < 0.01, and ^***^p < 0.001. As data for one patient at month 1 and for another patient at month 12 was only available for the condition “implant ON,” these data were excluded from the Wilcoxon analysis, but included in the summary statistics (mean + *SD*).

Results for the ADL task are summarized in Figure 2, showing the mean and the standard deviation (SD) for each group at each month and for each condition. Please note that for one patient at month 1 and for another patient at month 12, these assessments were only performed with the implant ON and not with the implant OFF. These patients were included in the summary statistics (mean, SD), but excluded from the statistical analysis with the Wilcoxon test as a pair-wise comparison for these patients would not have been possible.

#### Activities of daily living tasks—shapes

The pair-wise comparison of scores with implant ON vs. implant OFF showed that detection (“how many”) and localization (“where”) of geometric shapes was significantly better at all-time points, when the implant was switched on (see Figures 2A,B). Recognition scores (“what”) did not show statistically significant differences at any time point between implant switched ON and OFF (see Figure 2C).

#### Activities of daily living tasks—table

The pair-wise comparison of scores with implant ON vs. implant OFF showed that detection (“how many”) and localization (“where”) of table objects was significantly better at all-time points when the implant was switched ON (see Figures 2D,E). Recognition scores (“what”) were significantly better at month 3 only when the implant was switched ON as compared to OFF (see Figure 2F).

#### Activities of daily living tasks—eye-hand coordination

**Figure 3:**
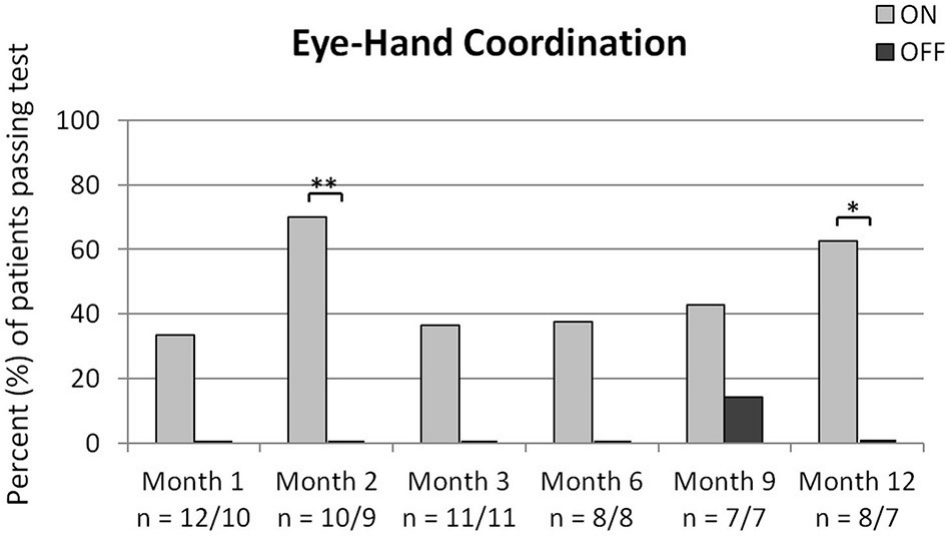
Shown are the percentages of patients passing the Eye-Hand Coordination test successfully at each time-point. Significant differences as analyzed via the 2-sided Fisher's exact test are shown in the figures with ^*^p < 0.05 and ^**^p < 0.01. The number of patients performing the test is indicated below the x-axis for implant ON/implant OFF. Except for one patient at month 9, none of the patients was able to position the object correctly when the implant was switched OFF.

The percentage of patients who were able to correctly position the object into the target region when the implant was switched ON was significantly greater at months 2 and 12 (see Figure 3). One patient (14.3%) passed the test successfully at month 9 with the implant switched OFF. Note the number of patients participating varies for the two conditions as two patients at month 1 and one patient each at month 2 and 12 did not pass the test with the implant switched ON and did not perform the test again with the implant switched OFF.

### Recognition of grayscales

**Figure 4:**
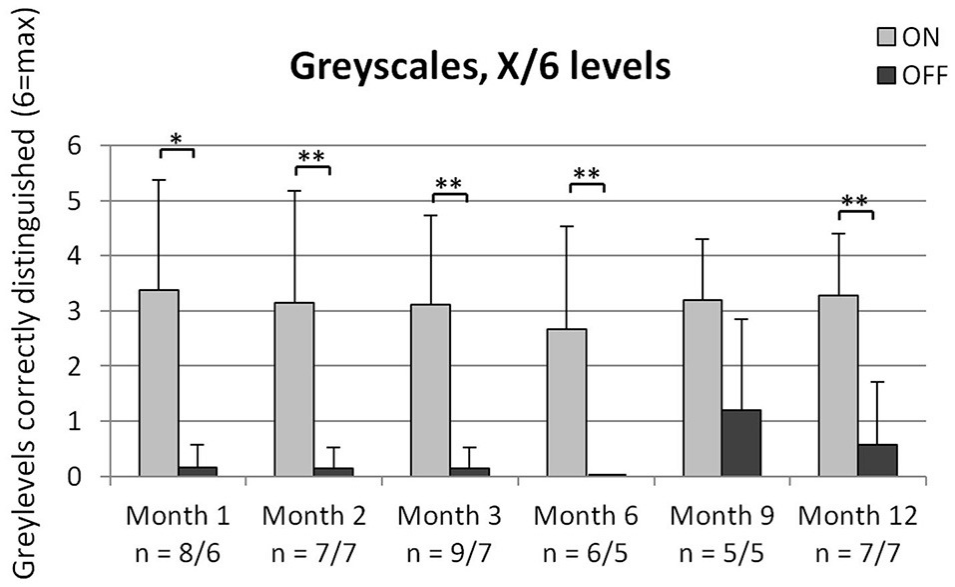
Shown are the mean + *SD* of correctly distinguished gray levels out of 6 combinations tested. The number of patients performing the test is indicated below the x-axis for implant ON/implant OFF. Significant differences as analyzed via the Wilcoxon test are shown in the figure with ^*^p < 0.05 and ^**^p < 0.01. As data of 2 patients at month 1 and 3 and of 1 patient at month 6 for was only available for the condition “implant ON,” these data were excluded from the Wilcoxon analysis, but included in the summary statistics (mean + *SD*).

As can be seen in Figure 4, patients performed significantly better when the implant was switched ON, compared to OFF, at months 1, 2, 3, 6, and 12. The recognition rate of the grayscales was partially dependent on the contrast. The combinations 50/0% (gray/white), 50/100% (gray/black) as well as 50/25% had the highest correct recognition rates.

### Basic light and motion test

**Figure 5:**
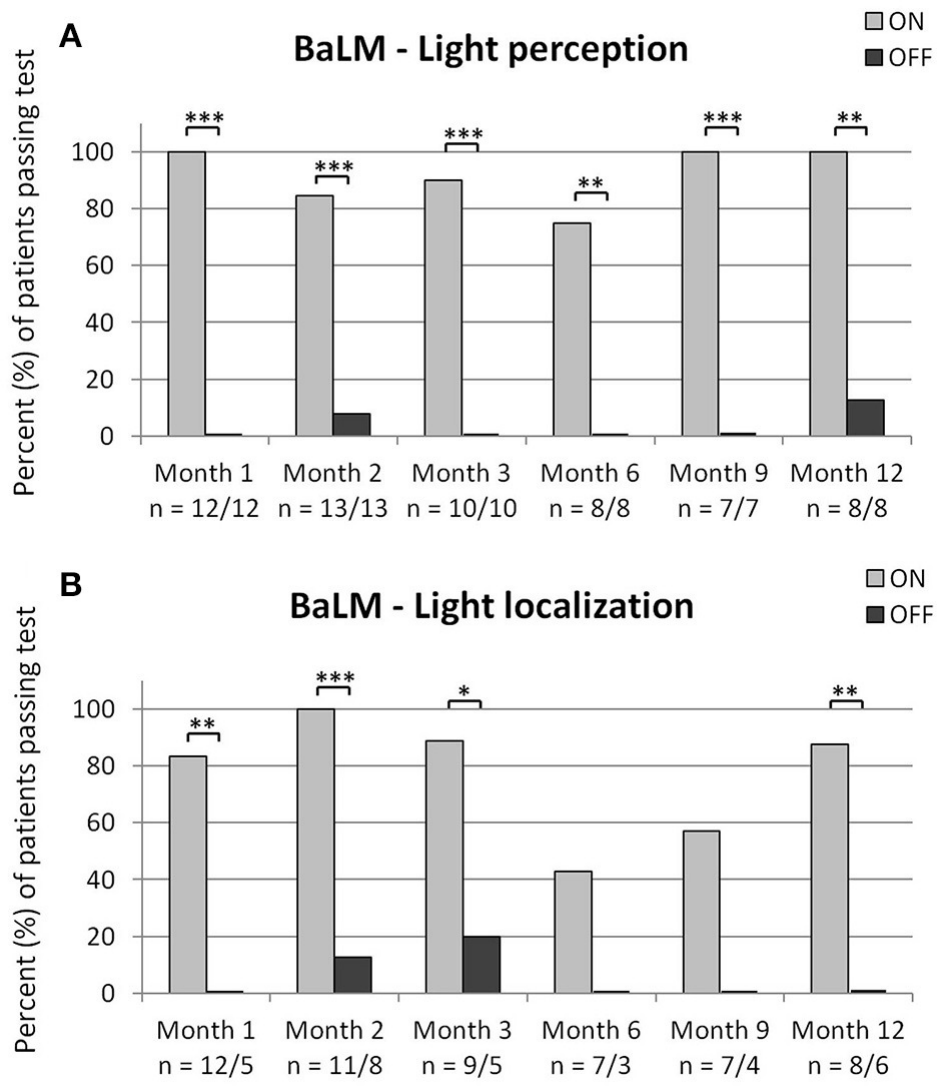
Shown are the percentages of patients passing the BaLM light perception test **(A)** and the BaLM light localization test **(B)** successfully at each time-point. Significant differences as analyzed via the 2-sided Fisher's exact test are shown in the figures with ^*^p < 0.05, ^**^p < 0.01, and ^***^p < 0.001. The number of patients performing the test is indicated below the x-axis for implant ON/implant OFF.

As can be seen in Figure 5A, light perception was possible in the majority of patients only when the implant was switched ON, which is reflected by the significant difference at all time points. Light localization was possible for most of the patients and was significantly better with the implant switched ON, as compared to OFF, at months 1, 2, 3, and 12 (see Figure 5B). Even though light perception was possible for 75% of patients at month 6 and 100% at month 9, only 43 and 57%, respectively were able to localize the light correctly. As can be seen in Figures 5A,B, the number of patients performing the tests of light perception and light localization varied for the condition implant ON. At month 2, 11/13 patients passed the light perception test. The two patients who did not pass this test were not subjected to the light localization test. One patient at month 3 was not subjected to the light localization test even though he passed the light perception test (joint decision between patient and investigator). If a patient failed the light localization test with the implant ON, a repetition of the test with the implant OFF was not obligatory, thus the lower number of patients performing the tests with implant OFF.

Motion detection was possible for two out of 11 patients when the implant was switched ON at month 1. No patient passed this test with the implant switched OFF at any time point (data not shown).

### Basic grating acuity and visual acuity

**Figure 6:**
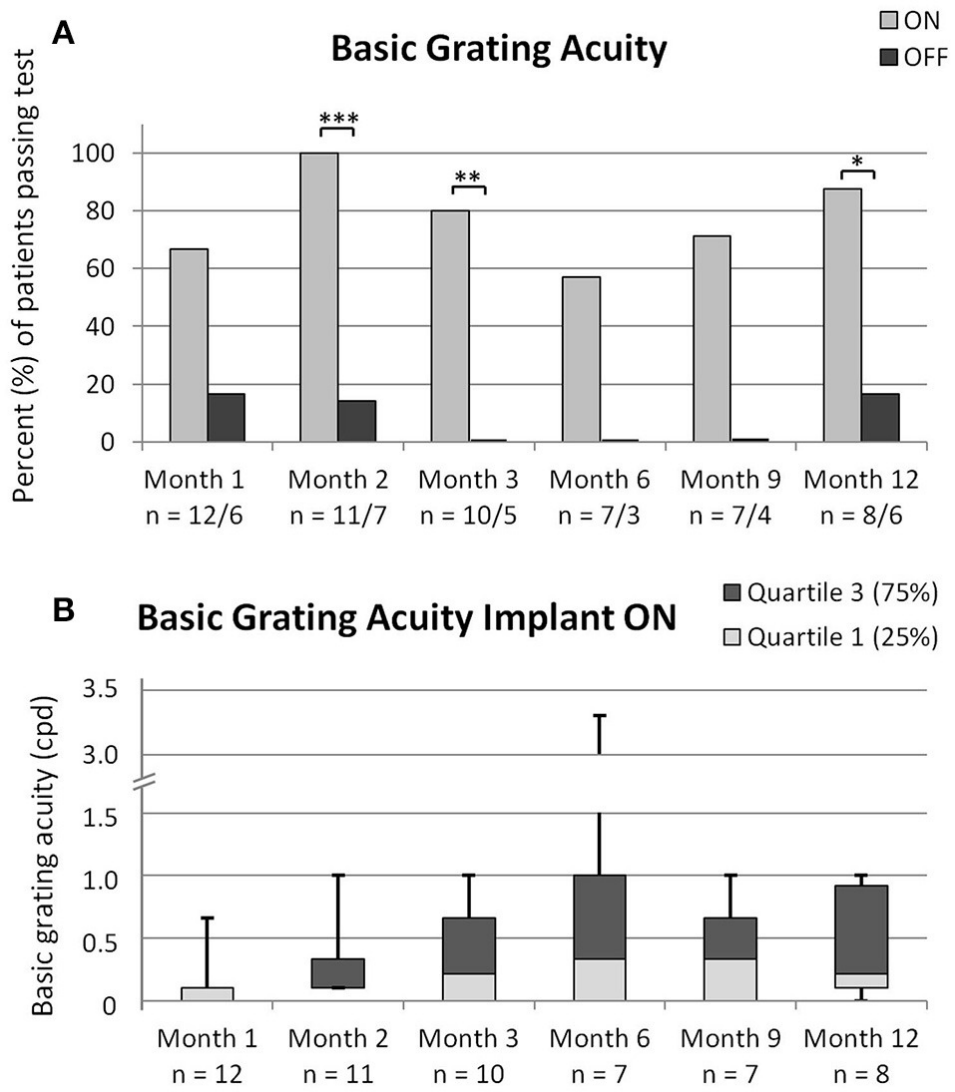
**(A)** shows the percentages of patients passing the basic grating acuity test successfully at each time-point for the conditions implant ON and implant OFF with significant differences as analyzed via the 2-sided Fisher's exact test shown in the figures with ^*^p < 0.05, ^**^p < 0.01, and ^***^p < 0.001. The number of patients performing the test is indicated below the x-axis for implant ON/implant OFF. **(B)** shows the data of patients in cycles per degree (cpd) with implant ON with the bottom white and top gray box representing the first and third quartile, and the band inside the box representing the median. The ends of the whiskers represent the minimum and maximum values. For failed tests, a value of “0 cpd” was noted. Please note the broken y-axis. The number of patients performing the test is indicated below the x-axis.

Detection of grating orientation was significantly better with the implant switched ON compared to OFF at month 2, 3, and 12 (see Figure 6A). Even though more than half of the patients passed the test at the other time-points, the differences to implant OFF were not significant; which is probably due to the low number of data points for the condition implant OFF. As can be seen in Figure 6B, median basic grating acuity (in cycles per degree) with implant ON ranged from a median of 0.1 at month 1 and 2 to 0.33 at month 6 and 9 (the median at month 3 and 12 was 0.215).

Visual acuity measurement with Landolt C-rings was possible in two patients with one patient reaching 20/1111 and the other patient 20/546 Snellen visual acuity.

## Discussion

The results presented in this paper demonstrate that the subretinal implant RETINA IMPLANT Alpha AMS is able to restore vision to a limited extent in patients with end-stage retinal dystrophy. The Alpha AMS device is an improved version of the previously developed Alpha IMS device, for which results have previously been published (Stingl et al., 2015). Device changes as summarized in Table 1 included an increased amount of pixels and various iterations on the material properties and functional characteristics to improve the durability of the device, leading to an estimated median operating life of 3.3 years, based on clinical data and 4.7 years based on laboratory data from accelerated aging tests of the implant components (Daschner et al., 2017).

**Figure 7:**
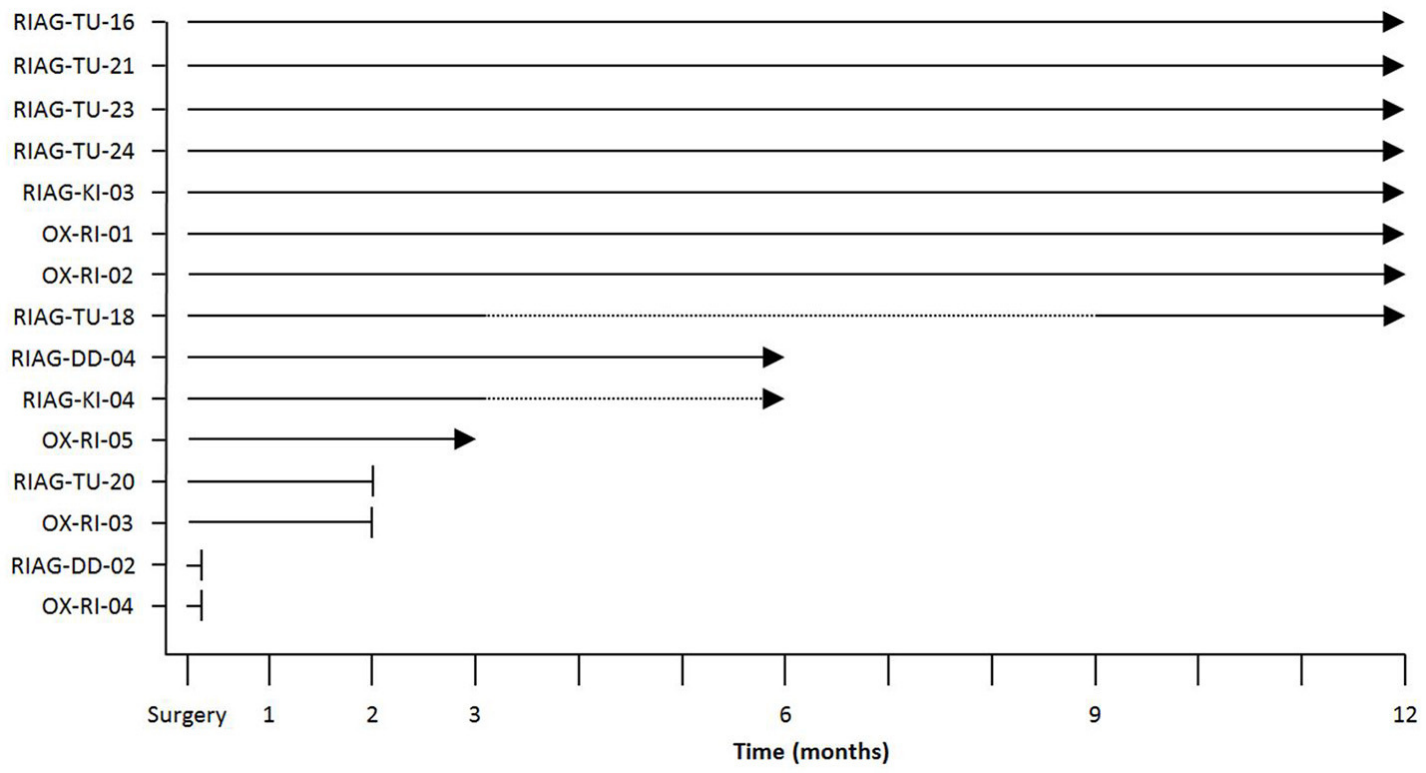
Overview of data available at the time of this report for the time points studied with solid lines indicating available data, dotted lines indicating that no visit occurred despite implant working properly (RIAG-TU-18, month 6 and 9; RIAG-KI-04, month 6) and arrowheads indicating a functioning implant. The blunt ends indicate that the implant stopped functioning; a detailed explanation for implant failures is given in the text.

The results from the new AMS device, presented within this paper, are based on interim results from patients at four sites, with data monitoring/collection to be completed for three patients (see Figure 7). For one of these patients (RIAG-DD-04), data is present up to month 6 and for two patients, data is available up to month 3 (OX-RI-05, RIAG-KI-04) although the latter did not attend the visit at month 6. Eight patients completed the study with one patient (RIAG-TU-18) not attending visits at month 6 and 9. In patients RIAG-TU-20 and OX-RI-03 the implant stopped working after 2 months, due to technical failures. Patient RIAG-DD-02 reported no reproducible visual perception with the implant. Examination of the implant after explanation revealed a small damaged region of the PI-foil probably due to mechanical impact of a needle during the implantation procedure. In another patient (OX-RI-04) a combination of damage to the connecting foil and incorrect implantation procedure resulted in a non-functional chip. The chip was eventually explanted and replaced with a new one which has since had “normal” function. Examination of the explanted chip confirmed that damage had occurred to the edge of the connecting foil and was most likely related to a forceps injury sustained whilst gripping the foil during the surgical implantation steps. There was no sign of any other technical problem with the chip itself. Further refinements to the surgical technique have been applied to avoid this complication in the future.

Despite the technical advances in the last couple of years, no device intended to restore vision in blind patients is able to provide the same spatial resolution as compared to a healthy eye. Preclinical work (Stett et al., 2000, 2007) has shown that electrical stimuli presented to the retina with a distance closer than 50 μm will not be spatially resolved with the present electrode configurations. For the RETINA IMPLANT Alpha AMS, estimations based on the reduced eye model result in a theoretical maximal possible visual acuity for two point discrimination of ~20/280.

In our cohort of 15 patients, two patients were able to distinguish Landolt C-rings up to 20/1111 and 20/546. These two patients were also able to discriminate the orientation of gratings with a spatial frequency of 0.66 and 1 cpd, respectively. These values are in line with the results from patients using the previous Alpha IMS device, where the maximum value of visual acuity achieved was 20/546 in one patient. For both types of implants the Landoldt C-ring visual acuity was reproducible (achieved by both patients during at least two visits). Landolt C-ring measurements under regular ophthalmological conditions have only been reported for one other retinal prosthesis, namely the supra-choroidal device from Bionic Vision Australia, where one patient was able to achieve a maximum value of 20/4451 visual acuity (Ayton et al., 2014). Recognition of Landolt C-rings under regular ophthalmological conditions has not been reported for any type of epiretinal prosthesis so far, but grating visual acuity was reported to be 1.8 logMAR (Snellen 20/1260) and 1.9 logMAR (Snellen 20/1588) in two patients using the Argus II device (Humayun et al., 2012; Ho et al., 2015).

The best basic grating acuity (recognition of direction of a grating) achieved in our cohort was 3.3 cpd by one patient. This patient was not able to identify Landolt C-rings; supporting the wide-spread notion that correct recognition of orientation of lines in a four alternative forced choice test that involves sampling of many lines over a large area is not related to visual acuity determined by simple optotypes in very low vision. The result of 3.3 cpd has been achieved by one patient with the RETINA IMPLANT Alpha IMS as well (Stingl et al., 2015).

Implant-mediated visual perception was generally stable over the observation period of 12 months. The implant was activated 1 month after surgery (month 1), and as the data of all assessments is comparable to the later time-points the authors conclude that re-learning vision is not, or only to a very small extent, necessary for this subset of patients. The authors' empirical experience confirms that mostly within days up to 2 weeks after the first switch-on of the device the best achievable vision for the individual patient could be estimated based on the present results. The authors believe that this is one of the most important differences between bionic vision with subretinal and epiretinal devices; while a subretinal device only tries to replace the photoreceptor function, stimulating the remaining bipolar cell network, the vision is perceived more “naturally” because the whole remaining visual pathway is used. In contrary, in epiretinal visual devices, the third visual neuron is stimulated, bypassing the preprocessing of the bipolar cell layer. As the second visual neuron already performs important signal processing, a vision circumventing this natural step might require more learning and adaptation of the higher visual areas. The improved durability compared to the earlier version Alpha IMS is also contributing to the stable nature of the observations. As can be seen in Figure 2, the detection and localization scores of the objects do not show the tendency to decrease over the 12 month period, as it was the case for RETINA IMPLANT Alpha IMS which suffered from technical failures. However, recognition of items in the sense of shape perception and interpretation in the ADL tasks (Figures 2C,F) is more difficult for the patients than the detection (Figures 2A,D) and the localization (Figures 2B,E). This is caused by the simple fact, that the detection and localization of a white object on a dark background is possible even if there is a single flash perception while looking at the object, whereas for the object recognition a shape perception or even seeing of details is necessary. As the individual visual functions varied among the subjects, only a part of those who could localize the objects, were able to recognize the shapes.

The results from the ADL tasks show comparable mean scores as in the previously published cohort of 29 patients with RETINA IMPLANT Alpha IMS (Stingl et al., 2015).

Seeing motion with a retinal implant can be a very challenging task, depending on the stimulation frequency, the range of motion speed testing, and on the patient's remaining retinal processing abilities itself. Technical devices function with a working frequency corresponding to the stimulation frequency of the particular neurons. RETINA IMPLANT Alpha AMS had a working frequency of 1–10 Hz in our patients. Although 10 Hz produce to a rather continuous perception, lower frequencies lead to a kind of stroboscopic vision, making motion perception very difficult.

The eye-hand coordination task was included to check the performance of localization of the objects in coordination with hand movements, as the perception of directions may differ from the reality in blind patients. As fixation is lost in bilateral blindness, the brain creates an inner conception of the “straight forward” direction, which can lead to a shift in the localization perception after regaining vision. We indeed observed this phenomenon in some patients and noticed an inter-individually different but intra-individually consistent shift in the eye-hand coordination. Some trained patients were able to “integrate” the shift into their perception.

At the end of the observation period, seven out of eight patients were able to distinguish side-by-side comparisons of differing levels of gray from each other with the device turned ON (for one patient who reached that time-point, this assessment was not done at the last visit) compared to two out of eight who could while the device was turned OFF (5 patients failed the test and one patient did not perform this test). Similar results were obtained in the BaLM (Basic Light and Motion) tests, where eight out of eight patients completing the study had light perception with the implant ON compared to only one patient with the implant OFF. Seven out of these eight patients could also locate the light source correctly with the implant ON, but none of them was able to pass the motion test at month 12. Interestingly, seven patients were able to pass the basic grating acuity test at this time-point. This might be due to the fact that the standardized motion test applied in this study can be regarded as more challenging to the visual system for these patients as it also relies heavily on the setting of stimulation frequency, motion speed range and eye movement. However, even if not passing the formal limited motion speed test range, many patients reported spontaneously about perceiving moving objects such as car lights, animals, persons etc. As the working frequency of the implant can differ from patient to patient and from visit to visit (between 1 and 10 Hz), the perception of motion is different for each patient and difficult to assess over the entire physiological range of motion perception.

The safety profile of the RETINA IMPLANT Alpha AMS shows four patients experiencing a total of eight Serious Adverse Events (SAEs). Postoperative movement of the implant was detected in two patients and was treated by repositioning the implant in a second surgery. Two patients experienced conjunctival dehiscence (one patient once and the other patient three times); all of them were treated by surgical intervention. One patient presented with a partial loss of silicone oil tamponade which was refilled in a second surgery. The same patient described pain in the region of the implanted ceramic housing in case of pressure a couple of months later. Initially, a surgical intervention was planned, but the patient declined and stated that the pain subsided. A surgical intervention for revision of the retroauricular cable positioning was finally done 10 months after the trial close-out visit (22 months after implantation). Adverse events were comparable to the safety profile described for the previous version Alpha IMS (Kitiratschky et al., 2015) and were mostly transient and of mild to moderate intensity. A detailed description of the safety profile of the RETINA IMPLANT Alpha AMS will be published after completion of these studies.

Overall, the new subretinal implant RETINA IMPLANT Alpha AMS has similar functional outcomes as the previous implant Alpha IMS, but with a considerably longer durability.

According to the European Directive 90/385/EEC extensive technical, pre-clinical and clinical documentation including a clinical evaluation (company internal document TD07K19_1) discussing interim results of the clinical trial NCT01024803 was submitted to the notified body (ID 0197) in Germany. Based on this documentation, RETINA IMPLANT Alpha AMS received the CE-mark in March 2016 from the notified body while the clinical trial was still continued for long term evaluation (Registration No. II 60119225 0001), according to European rules for medical devices. The Retina Implant Alpha AMS is now commercially available, with reimbursement by public health insurances at selected ophthalmological centers in Germany.

## Ethics statement

These studies were carried out in accordance with the recommendations of ICH Guidelines for Good Clinical Practice (CPMP/ICH/135/95) July 1996 with written informed consent from all subjects. All subjects gave written informed consent in accordance with the Declaration of Helsinki. The protocol was approved by the Ethics Committee of the Medical Faculty and University Hospital of Tuebingen, located in Gartenstrasse 47, 72074 Tuebingen, Germany and the National Research Ethics Service Committee London—Dulwich, Skipton House, 80 London Road, London SE1 6LH.

## Author contributions

Substantial contributions to the conception or design of the work: KS, KB, DB, FG, UG, RM, JRa, JRo, HS, NT, AR, and EZ. Substantial contributions to the acquisition of data for the work: KS, CC, TE, KK, LK, GS, JT, and EZ. Substantial contributions to the analysis and interpretation of data for the work: KS, RS, TE, NT, and EZ. Drafting the work or revising it critically for important intellectual content: All Authors. Final approval of the version to be published: All Authors. Agreement to be accountable for all aspects of the work in ensuring that questions related to the accuracy or integrity of any part of the work are appropriately investigated and resolved: All Authors.

### Conflict of interest statement

KS, LK: Employed by University of Tuebingen by means provided by Retina Implant AG, Reutlingen for the clinical trial, travel support. RS, UG, NT: Employees from Retina Implant AG, Reutlingen. FG, EZ: Stock ownership in Retina Implant AG, Reutlingen, paid consultant, holder of patents as inventor/developer, travel support from Retina Implant AG, Reutlingen. AR: Paid consultant, holder of patents as inventor/developer, travel support from Retina Implant AG, Reutlingen. HS: Stock ownership in Retina Implant AG, Reutlingen, paid consultants. JT: travel support from Retina Implant AG, Reutlingen. The other authors declare that the research was conducted in the absence of any commercial or financial relationships that could be construed as a potential conflict of interest.
